# Novel mTORC1 Inhibitors Kill Glioblastoma Stem Cells

**DOI:** 10.3390/ph13120419

**Published:** 2020-11-24

**Authors:** Jose A. Sandoval, Alexey Tomilov, Sandipan Datta, Sonia Allen, Robert O’Donnell, Thomas Sears, Kevin Woolard, Dmytro Kovalskyy, James M. Angelastro, Gino Cortopassi

**Affiliations:** 1 Department of Molecular Biosciences, University of California Davis, Davis, CA 95616, USA; jasandov@ucdavis.edu (J.A.S.); atomilov@ucdavis.edu (A.T.); sddatta@ucdavis.edu (S.D.); soniaallenphd@gmail.com (S.A.); jmangelastro@ucdavis.edu (J.M.A.); 2 Department of Internal Medicine, University of California Davis School of Medicine, Sacramento, CA 95817, USA; rtodonnell@ucdavis.edu; 3 Department of Pathology, Microbiology and Immunology, University of California Davis, Davis, CA 95616, USA; tksears@ucdavis.edu (T.S.); kdwoolard@ucdavis.edu (K.W.); 4 Department of Biochemistry, University of Texas Health and Science Center at San Antonio, San Antonio, TX, 78229, USA; kovalskyy@uthscsa.edu

**Keywords:** mTOR, mTORC1, glioblastoma, piperazine, meclizine

## Abstract

Glioblastoma (GBM) is an aggressive tumor of the brain, with an average post-diagnosis survival of 15 months. GBM stem cells (GBMSC) resist the standard-of-care therapy, temozolomide, and are considered a major contributor to tumor resistance. Mammalian target of rapamycin Complex 1 (mTORC1) regulates cell proliferation and has been shown by others to have reduced activity in GBMSC. We recently identified a novel chemical series of human-safe piperazine-based brain-penetrant mTORC1-specific inhibitors. We assayed the piperazine-mTOR binding strength by two biophysical measurements, biolayer interferometry and field-effect biosensing, and these confirmed each other and demonstrated a structure–activity relationship. As mTORC1 is altered in human GBMSC, and as mTORC1 inhibitors have been tested in previous GBM clinical trials, we tested the killing potency of the tightest-binding piperazines and observed that these were potent GBMSC killers. GBMSCs are resistant to the standard-of-care temozolomide therapy, but temozolomide supplemented with tight-binding piperazine meclizine and flunarizine greatly enhanced GBMSC death over temozolomide alone. Lastly, we investigated IDH1-mutated GBMSC mutations that are known to affect mitochondrial and mTORC1 metabolism, and the tight-binding meclizine provoked ‘synthetic lethality’ in IDH1-mutant GBMSCs. In other words, IDH1-mutated GBMSC showed greater sensitivity to the coadministration of temozolomide and meclizine. These data tend to support a novel clinical strategy for GBM, i.e., the co-administration of meclizine or flunarizine as adjuvant therapy in the treatment of GBM and IDH1-mutant GBM.

## 1. Introduction

Glioblastoma multiforme (GBM) is a deadly tumor with a usual post-diagnosis survival of 15 months. One major hypothesis for GBM tumor resistance is that GBM stem cells (GBMSCs) resist standard chemotherapy and, through growth, proliferation, and mutation, cause tumor relapse [1,2,3]. Several studies have shown that mTORC1 inhibition through rapamycin attenuates cancer stem cell chemotherapy insensitivity [4,5,6].

The mechanistic or mammalian target of rapamycin (mTOR) is a highly conserved protein kinase that serves as a central regulator of cell growth and connects cellular metabolism with environmental factors [7]. It is divided into two distinct complexes labelled mTORC1 and mTORC2, both of which are often dysregulated in cancer, including GBM [8,9,10]. mTORC1 is made up of a regulatory-associated-protein-of-mTOR (RAPTOR), proline-rich AKT substrate 40 kDa (PRAS40), mammalian lethal with Sec-13 protein 8 (mLST8), and DEP-domain TOR-binding protein (DEPTOR) [10]. It is inhibited by rapamycin, which binds to FK506-binding protein that only interacts with mTORC1. Growth factors, energy levels, oxygen levels, and amino acids all modulate mTORC1 activity through their effect on TSC1/2, a GTP-ase-activating protein that acts on Ras-homolog enriched in the brain (Rheb), which interacts with mTORC1. In the active form, mTORC1 promotes cell growth and proliferation mainly by phosphorylating eukaryotic translation initiation factor 4E binding protein 1 (4EBP1) and ribosomal protein S6Kinase (S6K). mTORC1 plays a significant role in autophagy, lipid synthesis, and mitochondrial metabolism and biogenesis, and is altered in many tumor types [11,12,13]. mTORC1 activity is altered in patient-derived GBMSCs [14], suggesting that inhibition of mTORC1 could be specifically lethal to GBMSCs.

Numerous mutations occur to drive GBM tumor progression. One such mutation is an R132H in the isocitrate dehydrogenase 1 (IDH1) protein. This is a gain of function mutation that alters metabolism, increasing the conversion of alpha-ketoglutarate into 2-hydroxyglutarate (D-2HG) [15]. Recently, it was shown that among patients with GBM IDH1 mutations, those patients with the lowest mTORC1 activity survived longest [16]. These authors found that among GBM patients with the IDH1 mutation, the low-mTORC1 group survived about 10-fold longer (200 months) than the high-mTORC1 group (20 months) [16]. Thus, it could be hypothesized that pharmacologically decreasing the mTORC1 activity in IDH1-mutant-bearing GBM patients could be a novel strategy for extending disease-free survival.

There are other connections between IDH1 and mTORC1. It was found that the 2HG metabolite produced in IDH1-mutant cells is an mTORC1 stimulator [17]. Others have observed that inhibition of mTORC1 reduces the 2HG metabolite that appears to underlie GBMSC progression [18]. Thus, in the IDH1-mutant > 2HG > mTORC1 > GBMSC progression pathway, a novel mTORC1 inhibitor might potentially slow progression, or be ‘synthetically lethal’ to IDH1-mutant tumors specifically.

Previously, we identified four piperazine compounds (cinnarizine, hydroxyzine, meclizine, and flunarizine) as novel mTORC1-specific inhibitors [19]. Two of the piperazine compounds, cinnarizine and flunarizine, have shown promise as radiation sensitizers in numerous mouse tumor types, perhaps through this previously unknown capacity to inhibit mTORC1 [20,21]. Given the finding of altered mTORC1 in patient-derived GBMSCs [14], we hypothesized that piperazine mTORC1 inhibitors might be effective killers of GBMSCs in vitro. Furthermore, because these drugs are known to be brain-penetrant and have low side-effect profiles, we tested the concept that they might be used as adjuvant therapy alongside the chemotherapeutic standard of care in GBM, namely temozolomide. Lastly, we tested the hypothesis that through the metabolic connection between IDH1 mutations and mTORC1, these mTORC1 inhibitors would be synthetically lethal to GBMSCs harboring the IDH1 mutation. Our results tend to support the ideas that piperazine toxicity to GBMSCs is related to their binding to the mTOR protein itself, that the piperazines plus temozolomide combination is much more toxic to GBMSCs than temozolomide alone, and that piperazines appear to have synthetic lethality in GBMSCs in the context of IDH1 mutations. 

## 2. Results

### 2.1. Piperazine Compounds Dose-Dependently Inhibit mTORC1 Downstream Target

**Figure 1:**
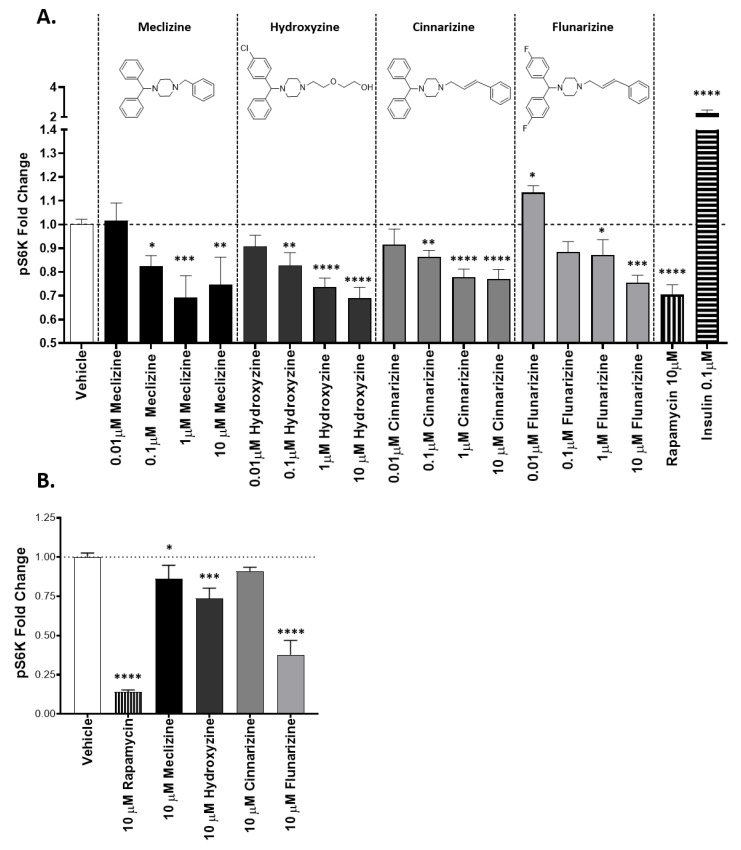
Confirmation of mTORC1 inhibition by piperazine compounds. (**A**) Piperazine drugs dose-dependently inhibit mTORC1 downstream target S6Kinase in mouse FL83B cells. (**B**) Piperazine drugs inhibit GBM stem cells at a single concentration of 10 µM. The amount of phosphorylated S6Kinase relative to total S6Kinase was measured by ELISA. The bars are the fold change over the vehicle control. Error bars are 1 standard deviation. Data shown for meclizine, hydroxyzine, cinnarizine, and flunarizine as indicated. *, **, ***, **** correspond to *p* values less than 0.05, 0.001, 0.0001, and 0.00001 respectively. *n* = 3 for each group. *p* value calculated with Student’s *t*-test.

Previously, our lab has shown that members of the piperazine drug class are mTORC1-specific inhibitors [19]. In Figure 1A, we show that four piperazines dose-dependently inhibit the downstream target of mTORC1, pS6K (Figure 1). Hydroxyzine and cinnarizine yield significant inhibition beginning at 0.1 µM and have a maximum effect at 10 µM. Meclizine and flunarizine follow similar trends, with meclizine showing significant inhibition beginning at 0.1 µM and flunarizine at 1 µM. Though meclizine does not show a maximum effect at the highest dose, all results are consistent with each other. In Figure 1B, we show that these four piperazine compounds at a single dose of 10 µM inhibit mTORC1, assessed through the phosphorylation of its downstream target pS6K, in patient-derived glioblastoma stem cells. Out of the four piperazine compounds, flunarizine exhibits the highest inhibitory potency, followed by hydroxyzine, meclizine, and cinnarizine, which shows an inhibitory trend.

### 2.2. Two Biophysical Assays Confirm Piperazine Interaction with Human mTOR

**Figure 2:**
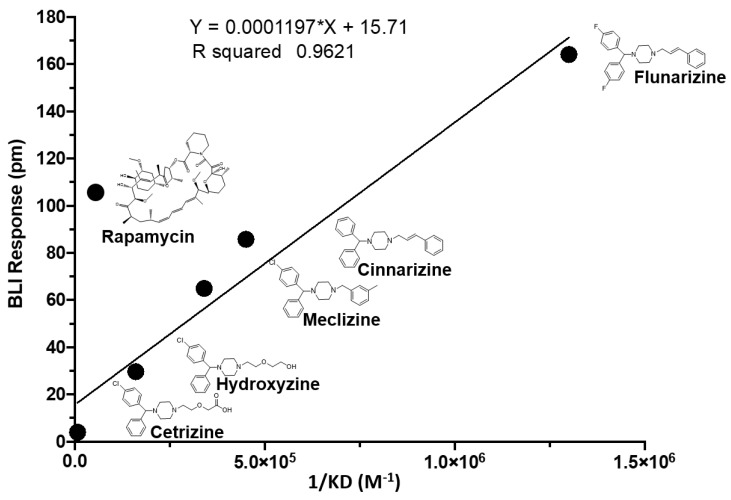
Affinity plotted against binding response for members of the piperazine drug class and rapamycin. The Y-axis is the response measured with bi-layer interferometry. Affinity measurements generated with field-effect biosensing is on the X-axis, as indicated. The line is linear regression.

In order to better-characterize the piperazine compounds and move forward in our selection of a top cancer therapeutic candidate, we measured the absolute response of small molecules binding to the human mTOR protein with bio-layer interferometry (BLI). As a further confirmation, we plotted the BLI response (pm) against the affinity expressed as the reciprocal of the dissociation constant of the same interactions as determined by field-effect biosensing (FEB). A linear relationship was observed. Flunarizine yielded the strongest response and highest affinity to human mTOR followed by cinnarizine, meclizine, hydroxyzine, and cetirizine (Figure 2). Rapamycin is included as a potent mTORC1 inhibitor; however, rapamycin has a different molecular weight, optical density, and charge-to-mass ratio that limits the integrity of comparing it to the piperazine compounds in these two biophysical assays. 

### 2.3. Piperazine Drugs Kill GBMSCs

**Figure 3:**
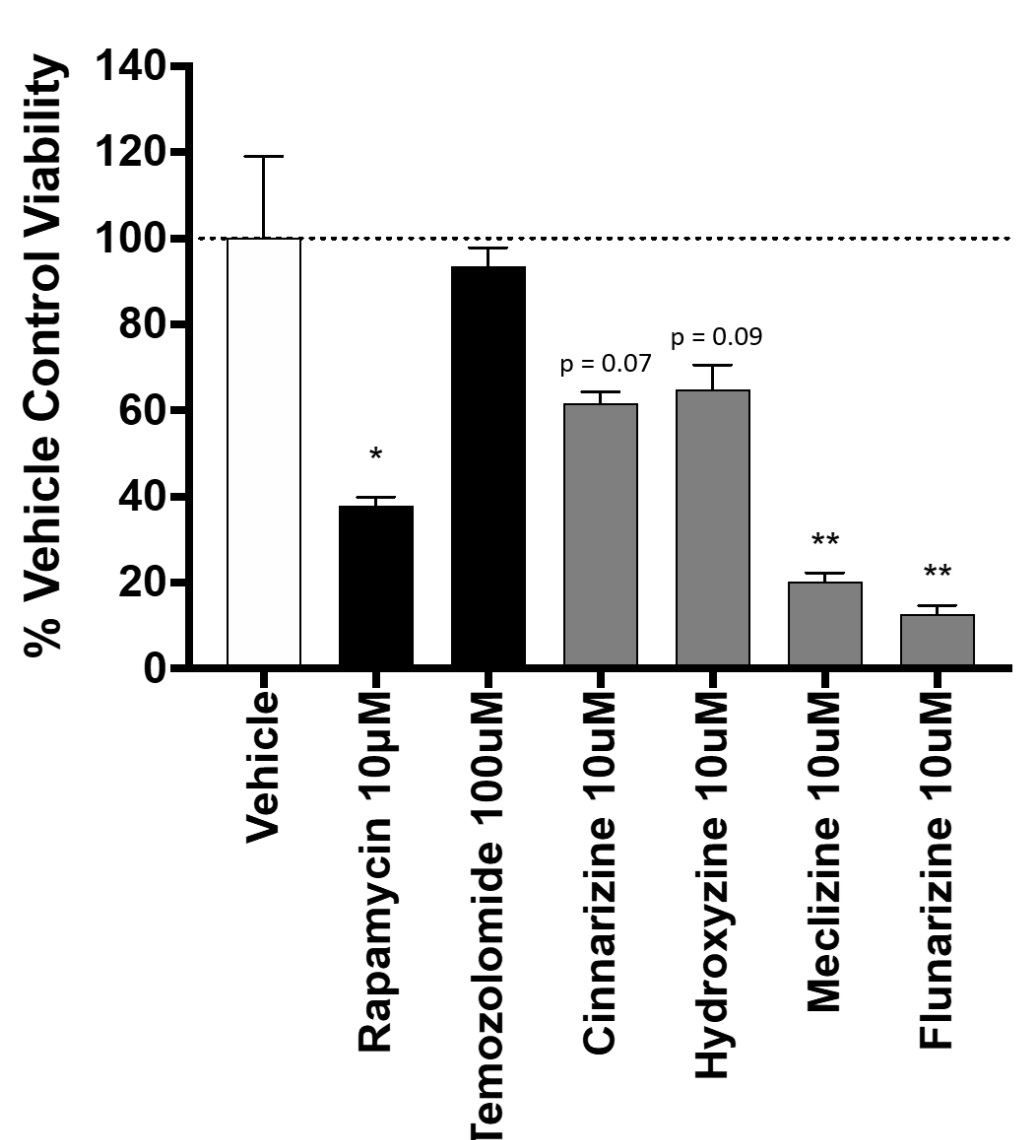
Piperazine drugs reduce viability in the patient-derived GBMSC line. Experiments were completed used Trypan Blue Exclusion. The columns are the average percent viability of three biological replicates normalized to the vehicle control. Error bars are the standard error of the mean. *, **: *p* < 0.05, *p* < 0.001, respectively. Compound names and concentrations are indicated. *p*-values calculated using the two-tailed Student’s *t*-test. *n* = 3 for compounds tested. As stated above, *p* values for cinnarizine and hydroxyzine were 0.07 and 0.09, respectively. *p* values for meclizine and flunarizine were 0.0082 and 0.0057, respectively.

The killing potency of four mTORC1-inhibiting piperazine compounds was tested (Figure 3). Killing was compared to rapamycin, the classic mTORC1 inhibitor, as well as temozolomide, the current standard of care for GBM. All four of the tested piperazine compounds reduced cell viability compared to either vehicle control or temozolomide. Meclizine and flunarizine were more toxic to GBMSC than rapamycin (Figure 3). Rapamycin has been used in multiple GBM clinical trials [22,23,24,25].

### 2.4. Comparison of GBM Cell Viability after Treatment with Meclizine, Flunarizine, or Rapamycin

**Figure 4:**
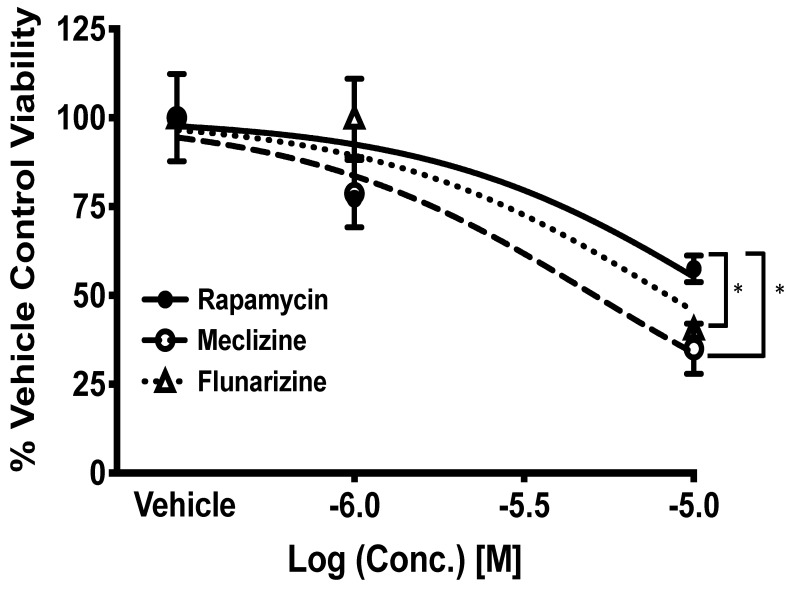
Meclizine and flunarizine dose-dependently kill patient-derived GBMSC better than rapamycin. Experiments were completed used Trypan Blue Exclusion. Values are cell viability at 1 µM (−6.0) and 10 µM (−5.0) of rapamycin, meclizine, and flunarizine. Error bars are the standard error of the mean. A significant difference for meclizine v. rapamycin and flunarizine v. rapamycin at 10 µM *p* < 0.05 was observed, this is represented by *. *p*-values were calculated using the two-tailed *t*-test. Lines are nonlinear sigmoidal dose–response curves fitted by the three-parameter model. *n* = 6 for vehicle, *n* = 4 for each concentration rapamycin, *n* = 6 for each concentration of meclizine, and *n* = 3 for each concentration of flunarizine. The total number of measurements was 6, 8, 12, and 6 for vehicle, rapamycin, meclizine, and flunarizine, respectively.

Meclizine, flunarizine, and rapamycin were tested in GBMSCs at four doses. Meclizine reduced GBMSC viability by nearly 70% at 10 µM. Flunarizine reduced GBMSC viability to a similar extent (Figure 4). Both meclizine and flunarizine were significantly more toxic to GBMSCs than rapamycin, a drug that has been used in several GBM clinical trials [22,23,24,25]. The IC_50_’s for meclizine, flunarizine, and rapamycin were 5.3, 6.8, and 14 µM, respectively (Figure 4).

### 2.5. Co-Administration of Temozolomide with Meclizine or Flunarizine Potentiates GBMSC Killing

Temozolomide is the standard-of-care chemotherapeutic agent used in GBM [9]. Temozolomide is used daily for 6 weeks during radiation therapy, and then again after a one-month break, for a further 6 months, and is given for 5 days per month. 

**Figure 5:**
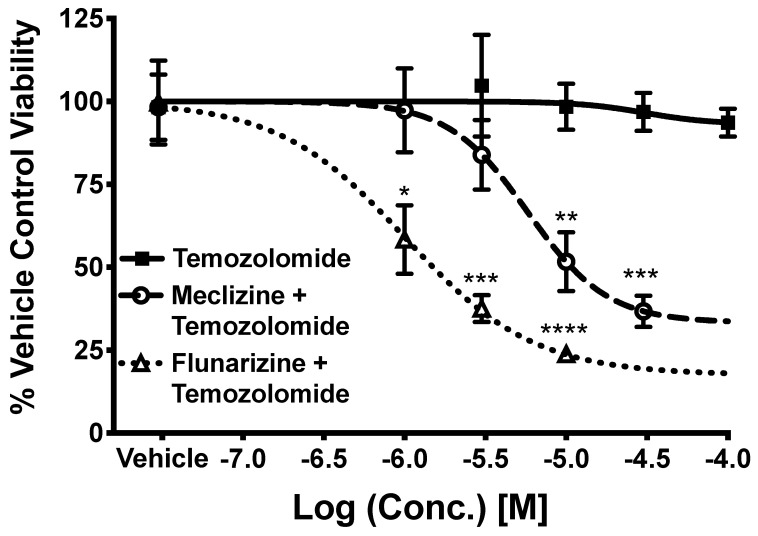
Meclizine and flunarizine co-administered with temozolomide, the current standard of care for GBM. Experiments were conducted using Trypan Blue Exclusion in patient-derived GBMsc. Experimental points are the average of 3 experiments. Error bars are the standard error of the mean. The curve is a four-parameter-model sigmoidal dose–response curve. The X-axis is drug concentration, while the Y-axis is % viability of GBSC normalized to vehicle control. Best-fitting values for the co-administration of meclizine and temozolomide were: IC50 = 5.7 µM, Rmin = 33.27, R2 = 0.99, *p* = 7.5 × 10^−8^. Best fitting values for flunarizine and temozolomide were: IC50 = 1.0 µM, Rmin = 17.50, R2 = 0.99, *p* = 4.5 × 10^−5^. Values for temozolomide alone: IC50 = 3.2 × 10^−5^, Rmin = 93.2, R2 = 0.71, *p*-value = 3.5 × 10^−2^. *n* = 4 for vehicle, 3 for each concentration of temozolomide, 3 for each concentration of meclizine + temozolomide, 3 for each concentration flunarizine + temozolomide. The total number of measurements are 3, 12, 12, and 9 for vehicle, temozolomide, meclizine + temozolomide, and flunarizine + temozolomide, respectively. *, **, ***, and **** correspond to *p* values less than 0.05, 0.001, 0.0001, and 0.00001 respectively.

Because meclizine has a benign safety profile (one tradename is Dramamine) [26,27,28,29], we tested the idea of meclizine as adjuvant therapy, i.e., the ability of piperazine plus temozolomide to kill GBMSCs. As seen in Figure 5, there was a negligible decrease in GBMSC viability at all doses of temozolomide. Even at 100 µM of temozolomide, the cell viability remained above 90%. The four-parameter sigmoidal dose–response model used for nonlinear regression predicted the survival of the cells (Rmin) to be 93.15%. This means that at any pharmacologically relevant concentrations of temozolomide treatment alone, there will be no more than a 6.85% reduction in viability of the GBMSC. In other words, the maximum killing potency of temozolomide alone is 6.85% for the GBMSC. Thus, temozolomide alone is not sufficient to combat GBMSC in vitro. 

Therefore, we tested our top candidates, flunarizine and meclizine, in combination with temozolomide, and observed dose-dependent killing (Figure 5). The tightest mTOR-binding compound, flunarizine, plus temozolomide appeared to be the most potent killing combination. The IC_50_ of flunarizine + temozolomide was 1 µM and the maximum cell killing potency in this experiment was 82.5%, *p* = 4.5 × 10^−5^. For meclizine + temozolomide, the IC_50_ was 5.7 µM and the maximum killing potency was 66.7%, R^2^ = 99, *p* = 7.5 × 10^−8^. Thus, the maximum killing potency of meclizine in combination with temozolomide was 66.7/6.85% = 9.6 times better than TMZ alone; the maximum killing potency of flunarizine in combination with temozolomide was 82.5%/6.85% = 12 times better than TMZ alone (maximum killing potency = 100-R/min). 

### 2.6. Meclizine Confers Synthetic Lethality on GBM Stem Cell Harboring R132H IDH1 Mutation

**Figure 6:**
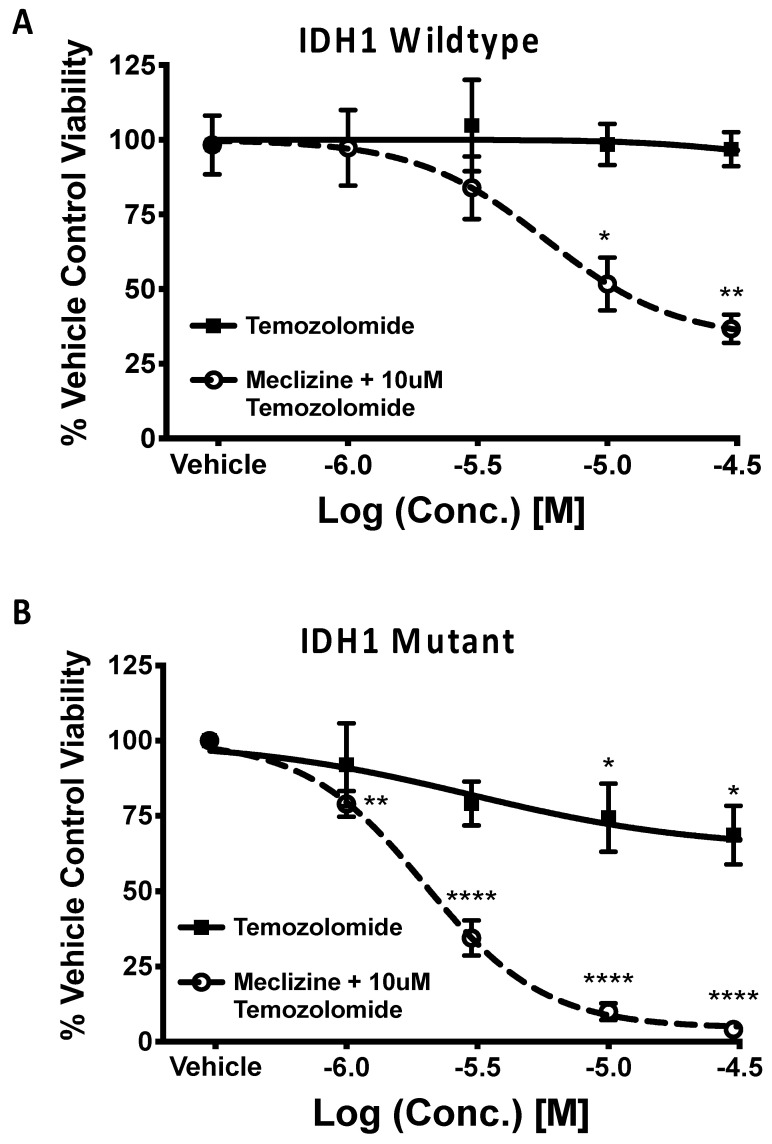
Comparison of cell viability reduction by temozolomide and by co-administration of meclizine and 10 µM temozolomide to the IDH1 wildtype (**A**), and in IDH1 R132H mutant (**B**) GBM patient-derived stem cells. The X-axis is the concentration of compounds tested. When meclizine was co-administered, the concentration of temozolomide was fixed at 10 µM. Experimental points are the average of 3 independent experiments; error bars are the standard error of the mean. The lines are the 4-parameter-model sigmoidal dose–response curves. *, **, **** *p* < 0.05, 0.01, 0.001, 0.0001, respectively. Best-fitting values for meclizine + temozolomide co-administration in wildtype (**A**) were: IC50 = 5.7 µM, Rmin = 33.27, R2 = 0.99, *p* = 7.5 × 10^−8^. *n* = 4 for vehicle, *n* = 3 for each concentration of meclizine + temozolomide. Best-fit values for temozolomide alone in wildtype (**A**) were: IC50 = 3.2 × 10^−5^, Rmin = 93.2, R2 = 0.71, *p*-value = 3.5 × 10^−2^. *n* = 4 for vehicle, and 3 for each concentration of temozolomide for a total of 12 concentrations of temozolomide. Best-fit values for meclizine co-administered in mutant (**B**) were: IC50 = 1.9 µM, Rmin = 4.1%, R2 = 0.99, *p*-value = 2.0 × 10^−6^. *n* = 10 for vehicle. *n* = 4 for each concentration of meclizine + temozolomide for a total of 16 measurements. Best-fit values for temozolomide in mutant (**B**) were: IC50 = 3.3 × 10^−6^, Rmin = 62.5%, R2 = 0.982, *p*-value = 6.1 × 10^−5^. *n* = 1 for vehicle. *n* = 6 for each concentration of temozolomide (24 measurements in total).

**Figure A1:**
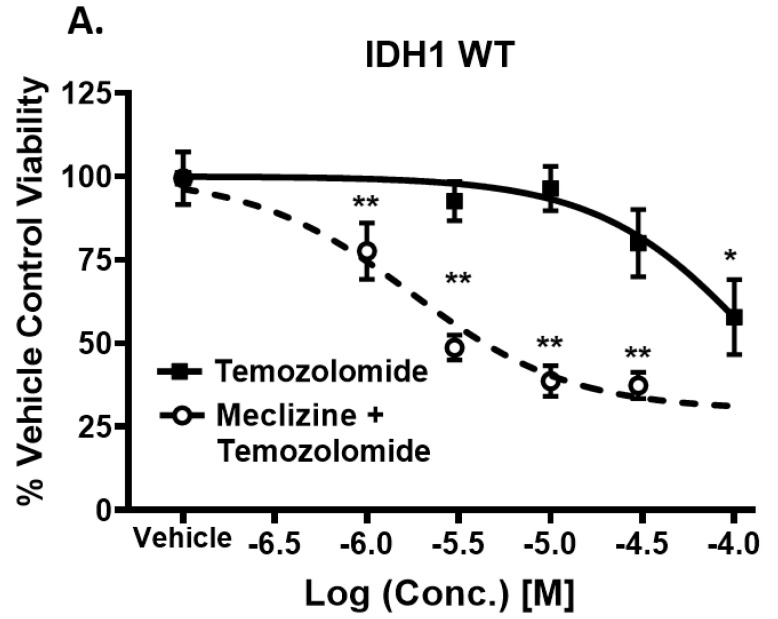

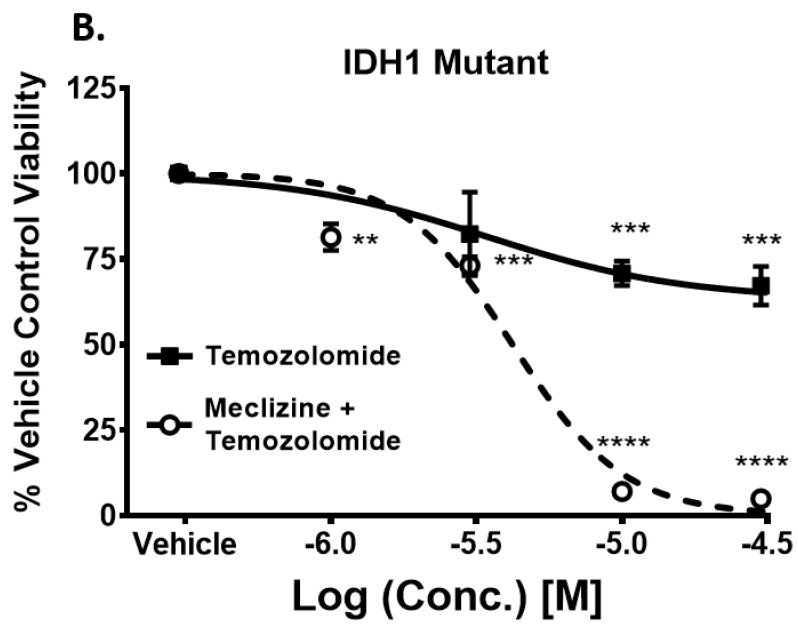
Coadministration of temozolomide and meclizine is more toxic than temozolomide alone. (**A**) Temozolomide and meclizine co-administered with temozolomide, the current standard of care, in a subsequent GBM IDH1 wildtype cell line and a (**B**) IDH1 mutant World Health Organization (WHO)-grade III astrocytoma. Experiments were conducted using Trypan Blue Exclusion in patient-derived GBMsc. Experimental points are an average of 3 experiments. Error bars are the standard error of the mean. The curve is a 3-parameter-model sigmoidal dose–response curve. The X-axis is drug concentration, while the Y-axis is % viability of GBMsc normalized to vehicle control. * *p* < 0.05, ** *p* < 0.01, *** *p* < 0.001, **** *p* < 0.0001.

GBM IDH1 mutations change metabolism and drive mTORC1 activity [17], and patients with GBMs and the IDH1 mutation have low mTORC1 activity and a 10-fold longer survival [16]. Thus, we tested the idea that mTORC1 inhibition via meclizine might cause synthetic lethality in IDH1 mutant cells. Figure 6A shows the IDH1 wildtype response to both temozolomide (black line) and meclizine + 10 µM temozolomide (dashed line). No significant difference in cell viability between the vehicle control and highest concentrations of temozolomide was observed. At higher concentrations, there was only about a 7% cell viability reduction observed in temozolomide alone. In experiments with the IDH1 wildtype cells, the co-administration of meclizine and temozolomide significantly decreased cell viability beginning at a 10 µM dose of meclizine. As stated above, the killing potency of meclizine in combination with temozolomide was about 67%, roughly 10 times better than temozolomide alone. Best-fit values for meclizine in combination with temozolomide were IC_50_: 5.7 µM, (100-Rmin): 66.7%, R^2^ = 0.99, *p* = 7.5 × 10^−8^, as determined by the four-parameter model. Figure 6B shows the R132H IDH1 mutant response. The IDH1 mutant appeared to be more sensitive to temozolomide alone than the IDH1 wild type, IC_50_ = 3.2 × 10 ^−6^, Rmin = 62.5% R^2^ = 0.97, *p*-value = 6.0 × 10^−5^; however, the maximum killing potency was only (100 − 62.5%) = 38%. While there was a significant reduction in cell viability at the two highest concentrations of temozolomide which are much higher than achieved in human dosing (30 and 100 µM), temozolomide alone is not very effective for killing GBMSC with the R132H genotype, as all cells survive at any pharmacologically relevant concentration of temozolomide alone. However, a significant reduction in cell viability was observed beginning at 1 µM of meclizine co-administered with 10 µM of temozolomide in the IDH1 mutant GBM stem cell line. Thus, the R1423 IDH1 mutant GBMSC was effectively and dose-dependently killed by the co-administration of meclizine with temozolomide. The IC_50_ of meclizine in combination with temozolomide was 2 µM, and the maximum killing potency was 95.5%. Therefore, the combination with meclizine was (95%/38%) >2 times more effective at killing the IDH1 GBMSC than temozolomide alone. Thus, the combination of meclizine with temozolomide might be an effective strategy to combat GBMSC, and especially the R132H mutant cells. Confirmatory dose-dependent killing was observed in another pair of IDH1 wt and mutant cell lines; the mutant in this case was a grade III astrocytoma rather than grade IV astrocytoma (Figure A1 in Appendix A). 

## 3. Discussion

**Figure A2:**
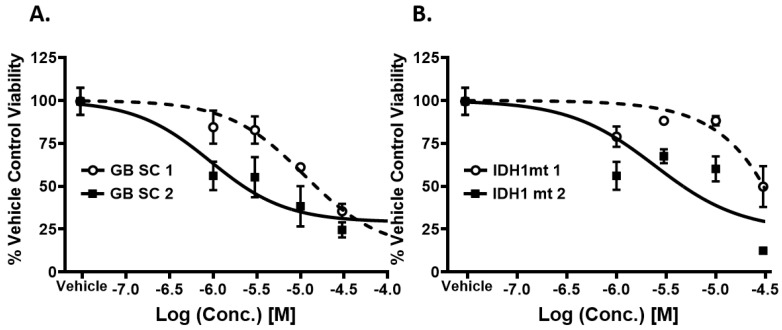
The mTORC1 inhibitor rapamycin is dose-dependently toxic to GBMSCs. (**A**) Rapamycin, also known mTORC1-specific inhibitor, dosed at increasing dosages in 2 patient-derived IDH1 wildtype GBMsc and in (**B**) 2 IDH1 mutant cell lines, with IDH1 mt 1 being a patient-derived GBMsc and IDH1 mt 2 a patient-derived WHO-grade III astrocytoma, which is known to develop into glioblastoma (WHO grade IV astrocytoma). Experiments were conducted using Trypan Blue Exclusion in patient-derived GBMsc. Experimental points are an average of 3 experiments. Error bars are the standard error of the mean. The curve is a 4-parameter-model sigmoidal dose–response curve. The X-axis is drug concentration, while the Y-axis is % viability of GBMsc normalized to vehicle control.

We recently demonstrated that a series of piperazine compounds are also mTORC1 inhibitors [19]. mTORC1 activity is a validated clinical target, and the mTORC1 inhibitor rapamycin, everolimus, and temsirolimus have been used in clinical trials of GBM [30,31,32]. Rapamycin, the most common mTORC1-specific inhibitor, has been approved for numerous clinical trials; however, rapamycin has an extensive side-effect profile [33]. This extensive side-effect profile may limit its effectiveness. Thus, the discovery of a novel class of mTORC1 inhibitors, such as meclizine with a lower toxicity profile than rapamycin, may be a relevant candidate for novel GBM therapy. As seen in Appendix A Figure A2, Rapamycin, through its mTORC1 inhibition, is an effective killer of 2 IDH1 wt GBMSC and of 1 IDH1 mutant GBMSC and another IDH1 mutant grade III astrocytoma stem cell line. In other words, we observe an increase in stem cell death with the increase in mTORC1 inhibition. This observation emphasizes the significance of novel mTORC1 inhibitors with milder safety profiles and similar potency to rapamycin as mTORC1 inhibition has shown benefits ranging from in vitro studies to human clinical trials [34,35].

Cinnarizine and flunarizine were two of the tightest binders to mTORC1, and were toxic to GBMSCs in our assays, having been shown by others to kill lymphomas and myeloma cells as well [36,37]. However, cinnarizine and flunarizine both have a chemical liability in that after prolonged exposure, they can cause iatrogenic parkinsonism [36,37,38], and are less likely to win FDA approval, even for a very severe cancer. Thus, we tested the potency of our tightest mTORC1-binders with milder side-effect profiles, meclizine, toward killing GBMSC. We also conducted these tests, though less extensive, on flunarizine as it was our tightest binder overall. Both meclizine and flunarizine performed better than rapamycin.

If a clinical trial were ever to be carried out using meclizine in GBM, ethical considerations would require it be co-administered alongside the standard-of-care drug temozolomide, as has been done with other mTORC1 clinical trials [24,39]. Meclizine and flunarizine when co-administered with temozolomide greatly increased cytotoxicity, suggesting them as possible adjuvant therapies with a low side-effect profile.

Meclizine is an antihistamine prescribed to treat nausea and motion sickness; flunarizine was previously prescribed to treat vertigo and migraines [40,41]. Due to meclizine’s mild safety profile, precedence in human dosing, and similar potency in reducing cell viability when compared to flunarizine and rapamycin, we propose meclizine as a potential GBM therapeutic agent. Additionally, meclizine has been well documented to cross the blood–brain barrier, an essential characteristic for treating GBM [42]. Nonetheless, we do not diminish the potential of flunarizine as a potential therapeutic agent as well. Further experiments in mouse models of GBM are necessary before implementing meclizine, or any other piperazine compound, as a cancer therapeutic. However, as safety and pharmacological studies have been extensively conducted, the timeline to clinical trial should be more straightforward than for a new chemical entity.

Additionally, we observed synthetic lethality when comparing the cell viability response of IDH1 wildtype GBMSCs and those bearing the R132H IDH1 mutation. Mutant cells have more 2-hydroxyglutarate, which mediates energy depletion and activated AMPK, which in turn, represses mTOR activity [43]. Seeing as alpha-ketoglutarate levels are reduced, indicating lower nutrient levels, a further decrease in mTORC1 through direct inhibition may be sufficient to remarkably reduce cell viability and proliferation when compared to IDH1 wildtype GBM stem cells. Elevated 2-HG levels also inhibit the DNA repair enzyme ALKBH and the DNA damage response proteins KDM4A/B [44,45]. Temozolomide, the standard-of-care chemotherapeutic agent for treating GBM, is a DNA alkylating agent, meaning that it targets GBM tumors by damaging DNA [46]. Clinically, IDH1 mutations are associated with a better response to temozolomide treatment, a fact supported by our temozolomide dose–response experiment in IDH1 mutant GBM cells (Figure 6B) [43,47]. The combination of an increased sensitivity to temozolomide and compound inhibition of mTORC1 by two means provide a molecular basis for IDH1 R132H synthetic lethality to the described treatment.

The need for additional cell lines is recognized as a limitation of the study. We have conducted less extensive studies on a subsequent IDH1 WT GBMSC labeled “GB SC2” in the appendix figures. In addition, we also conducted tests on an IDH1 mutant Grade III astrocytoma patient-derived cell line in the appendix figures. Though comparisons between Grade III astrocytoma and GBM, which is a grade IV astrocytoma, are not fair, results from the astrocytoma cell line are still interesting as Grade III astrocytoma develop into Glioblastoma. However, these experiments do confirm the benefit of the co-administration of meclizine with temozolomide in vitro and support their use as adjuvant therapy for GBM. In other words, meclizine co-administered with temozolomide killed more than temozolomide alone in three patient-derived GBM stem cell lines and in one grade III astrocytoma patient-derived cell line. Nonetheless, more primary cells harboring the IDH1 R132H mutation, either patient-derived or WT-derived, are necessary to sufficiently demonstrate that IDH1 mutant cells are more sensitive to mTORC1 inhibition than wildtype cells.

## 4. Materials and Methods

### 4.1. Cell Lines and Cell Culture

The Mus musculus (mouse) normal hepatocyte liver cell line (FL83B) was purchased from American Type Culture Collection (Manassas, VA, USA). FL83B cells were cultured in Dulbecco’s modified Eagle’s medium/Ham’s F-12 50/50 Mix with l-glutamine and 15 mM HEPES and 10% fetal bovine serum (FBS), both from Corning (Fremont, CA, USA). The cell line was grown at the standard 37 °C and 5% CO2 conditions. All drug treatment was performed in the absence of pen/strep and FBS.

**Figure A3:**
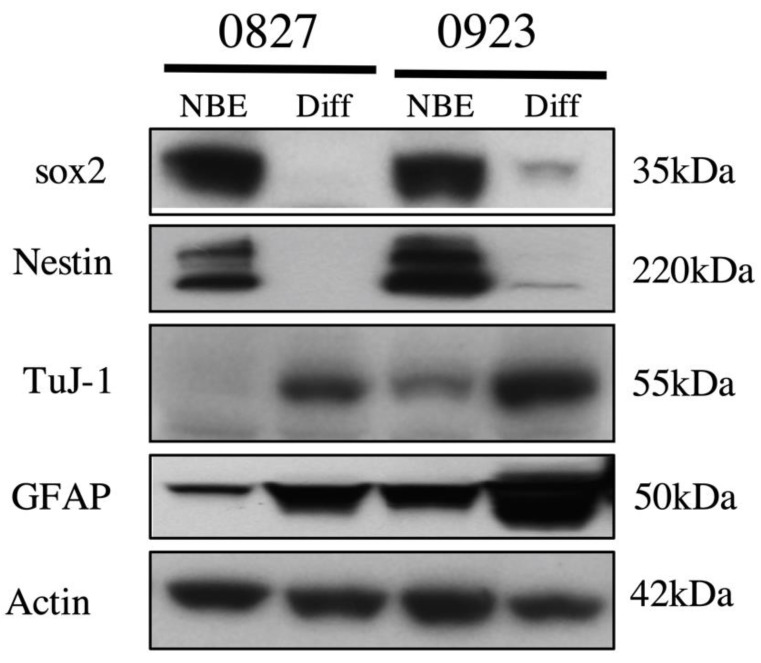
GBMSCs have appropriate stemness markers. Western Blot showing stemness of cells used in manuscript. 0827 and 0923 refer to IDH1 wt 1 and IDH1 wt 2, respectively. NBE refers to the cells in the “stem” state, and “Diff” refers to the cells after ten days of culture in media containing 10% FBS. Cells were in the “NBE” or “stem” state for all experiments presented in the manuscript. Molecular markers for stemness included are sox2 and Nestin. Markers of differentiation are TUJ-1 and GFAP. Actin served as the loading control.

The GBMSC lines used were patient-derived from recurring tumors and gifted to us by Kevin Woolard DVM, PhD from the University of California Davis. The cell lines were named 0827 and 0923. 0827 is the only IDH1 WT cell line used in the main figures in the manuscript and is referred to as IDH1 wildtype in Figure 6. 923 is used only in appendix and is referred to as “IDH1 WT” in Appendix A Figure A1 and “GB SC 2” in Appendix A Figure A2. The 0827 and 923 GBM stem cells were cultured in Neurobasal A minimal media with 1X N-2 supplement, 1X B-27 supplement, 1X GlutaMAX, and (50 units/mL of penicillin/50 μg/mL of streptomycin) from Gibco (Gaithersburg, MD, USA), and human EGF and FGF-154 from Shenandoah Biotechnology (Warwick, PA, USA). The IDH1 mutant cell line was named 905 and will be referred to as IDH1 mutant or R132H IDH1 mutant. It is the only IDH1 mutant cell line used in the main figures. The IDH1 mutant Grade III astrocytoma was named BT 142 and is only used in the appendix figures. It is referred to as “IDH1 mutant” in Appendix A Figure A1 and “IDH1 mt 2” in Appendix A Figure A2. These cells were cultured in the same media as the IDH1 wildtype with the addition of 1 mL of 10 µg/mL PDGF. Cells used did not exceed passage 15. Patient-derived glioma stem cells were generated at the National Cancer Institute, National Institutes of Health (Bethesda, MD). This research was supported by the Intramural Research Program of the NIH, National Cancer Institute, Center for Cancer Research and approved by the Institutional Review Board [48,49]. The stemness profile of IDH1 wildtype cells are included as a appendix figure (Figure A3). GBM-differentiated cells were cultured in Dulbecco’s modified Eagle’s medium (DMEM) supplemented with 10% FBS and pen/strep.

### 4.2. Compounds

All chemicals (DMSO, insulin, penicillin/streptomycin) were purchased from Sigma-Aldrich, unless indicated otherwise. Rapamycin was purchased from EMD Millipore (Billerica, MA, USA).

### 4.3. Bi-Layer Interferometry

Proteins we purified from HEK T293 cells. Terminally biotinylated mTOR protein and the GFP as a nonbinding control were produced using the following plasmids, for mTOR—EX Mm31144-M48, and for GFP—EX-EGFP-M48 GeneCopoeia (Rockville, MD, USA), and loaded onto sets of Octet RED 384 SSA biosensors (Pall ForteBio LLC., Menlo Park, CA, USA) to a density of 12 nm followed by the blocking of nonoccupied streptavidin residues of biosensors with 200 u biotin. Sensors were tested against indicated concentrations of compounds using the Octet RED 384 BLI instrument in BLI Kinetic Buffer (Pall ForteBio LLC., Menlo Park, CA, USA) containing 1% BSA. Parameters: Baseline 20 s, association 30 s, dissociation 40 s. Real-time binding sensorgrams were recorded and analyzed using Octet BLI software 8.1 (Pall ForteBio LLC., Menlo Park, CA, USA) and Excel.

### 4.4. Field-Effect Biosensing

The human 6His::mTOR was purified from HEK T293 cells using plasmid Ex-E1870-M01-GS GeneCopoeia (Rockville, MD, USA) and loaded onto the Ni-NTA chip Nanomedical Diagnostics (San Diego, CA, USA); compounds were tested at 10 µM concentration in triplicates; kon, koff, and KD were determined using Nanomedical Diagnostics Agile Plus Version 4.2.2.20754 software, Nanomedical Diagnostics (San Diego, CA, USA). The association time was 5 min, the dissociation time was 10 min, and the baseline was 15 min.

### 4.5. Cell Viability Assay

0827 IDH1 WT GBM, 923 IDH1 WT GBM, 905 IDH1 mutant GBM, and BT142 gIII astrocytoma stem cells were seeded, 300,000 cells/well, in 12-well cell culture plates and allowed to grow for 24 h. Cells were treated with vehicle (0.1% DMSO) or compounds dissolved in DMSO for 48 h before being assayed. Cells in media plus drug were then removed and centrifuged at 1200 rpm for 3 min. Media were aspirated off, and 0.5 mL of 0.05% trypsin was added to each well, incubated for 3 min, and then neutralized with serum containing media. Samples were re-suspended and pipetted thoroughly to create single cell suspensions for analysis. We utilized the Vi-CELLTM Cell Viability Analyzer from Beckman Coulter (Indianapolis, IN, USA) to evaluate cell viability and survival of both GBM stem cells after drug treatment.

### 4.6. pS6K ELISA

The PathScan^®^ Phospho-S6Kinase (Thr389) Sandwich ELISA and PathScan^®^ Total S6K Sandwich ELISA Kits were purchased from Cell Signaling Technologies (Danvers, MA). In addition, 200,000 cells/well were seeded onto 24-well plates and allowed to grow for 48 h; the media were changed to DMEM-F12 without FBS for the FL83B and standard Neurobasal for the Stem Cells, and cells were incubated for another 18 to 20 h. Cells were treated for two hours with tested compounds at indicated concentrations; the media were aspirated, and plates were washed once with ice-cold PBS. Cells were lysed with 200 μL of lysis buffer; lysates were transferred to a 96-well plate for ELISA. ELISA was performed according to manufacturer’s instructions.

### 4.7. Co-Immunoprecipitation

The HEK cells were co-transfected with N-terminally His-tagged Human mTOR Gene Copoeia Cat# EX-Mm31144-M48) and C-terminally Avi-tagged Human RAPTOR (Gene Copoeia Cat# EX-E0449-M62). The IP was performed with Ni-NTA magnetic beads (Thermo Fisher, Cat# 88831). Westerns were developed using IRDye^®^ 680RD Streptavidin (LiCor Biosciences, Cat# 926-68079) and anti-mTOR antibody, before the protein isolation cells were pre-treated with a 10 µM concentration of indicated drugs for 2 h. The bands intensities were quantified using Image Studio Software (Licor Biosciences Lincoln, Nebraska).

### 4.8. Western Blot

GBM cells were lysed using Cell Lysis buffer from Cell Signaling Technologies (Danvers, MA, USA) with 1X Halt™ Protease and Phosphatase Inhibitor Cocktail from ThermoFisher Scientific (Waltham, MA). Nitrocellulose membranes were incubated overnight with the following antibodies diluted in blocking buffer: 1:5000 dilution of anti-actin antibody, mouse monoclonal from Sigma-Aldrich, and 1:1000 Sox2 Rabbit mAb 1:1000; Nestin (Rat-401) Mouse mAb, β3-Tubulin (D71G9) XP^®^ Rabbit mAb, and GFAP (D1F4Q) XP^®^ Rabbit mAb all from Cell Signaling Technologies (Danvers, MA). Membranes were then incubated with the following secondary antibody pair: IRDye 680CW and IRDye 800CW-coupled secondary antibodies from LI-COR Biosciences (Lincoln, NE, USA) at 1:20,000 dilution. Western Blots were then visualized with the Odyssey infrared imager and software from LI-COR Biosciences (Lincoln, NE) according to the manufacturer’s instructions.

### 4.9. Data Analysis

Graphpad Prism 9.0 and Microsoft Excel were used for the statistical analysis. The three- or four-parameter sigmoidal dose–response model was used for fitting analysis (as indicated in legend). Significance was assigned as follows: * *p* < 0.05, ** *p* < 0.01, *** *p* < 0.001, **** *p* < 0.0001. All *p*-values were generated through Student’s *t*-test.

## Figures and Tables

**Figure A4:**
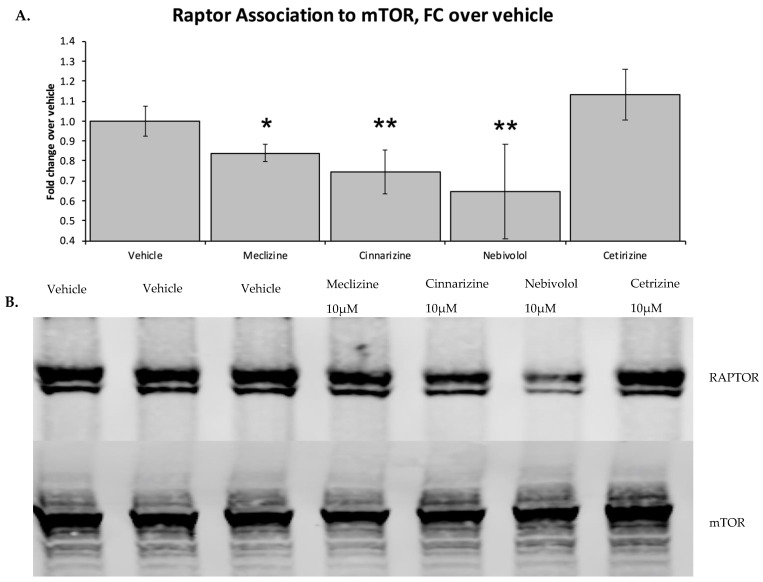
Piperazine molecules significantly block the association of Raptor with mTOR by co-immunoprecipitation. Co-immunoprecipitation showing mTOR and RAPTOR, component of mTORC1 complex. Experiment shows reduced loading of RAPTOR onto mTOR when treated with meclizine, cinnarizine, and nebivolol at 10 µM. This provides a potential mechanism for mTORC1-specific inhibition by piperazine compounds as RAPTOR is only associated with mTOR Complex 1. (**A**) The quantification of the gel, (**B**) bars are means and SD of 2 independent experiments. *p*-value calculated using two-tailed *t*-test. Meclizine, cinnarizine, and nebivolol were significantly inhibiting the association of mTOR with RAPTOR, but not cetirizine. Alpha was under 5%. * and ** refer to *p*-values less than 0.05 and 0.01 respectively.

**Figure A5:**
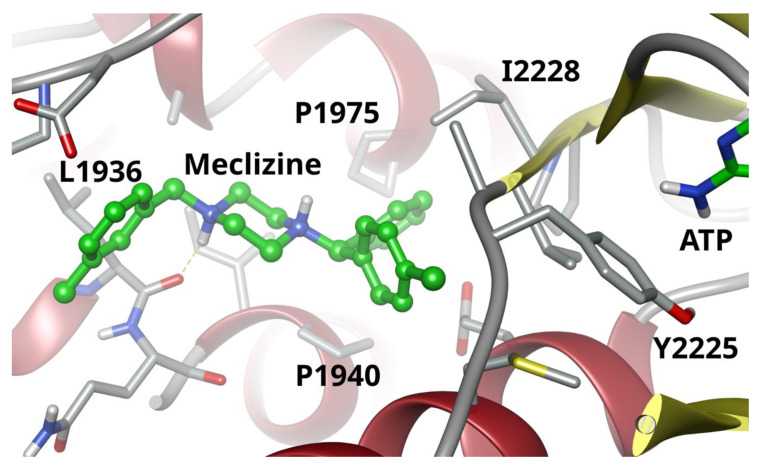
A pose of meclizine within the kinase domain of mTOR.
